# Effects of the Selective Stretch-Activated Channel Blocker GsMtx4 on Stretch-Induced Changes in Refractoriness in Isolated Rat Hearts and on Ventricular Premature Beats and Arrhythmias after Coronary Occlusion in Swine

**DOI:** 10.1371/journal.pone.0125753

**Published:** 2015-05-04

**Authors:** José A. Barrabés, Javier Inserte, Luis Agulló, Antonio Rodríguez-Sinovas, Juan J. Alburquerque-Béjar, David Garcia-Dorado

**Affiliations:** Servicio de Cardiología, Hospital Universitari Vall d’Hebron, Institut de Recerca (VHIR), Universitat Autònoma de Barcelona, Barcelona, Spain; University of California San Diego, UNITED STATES

## Abstract

Mechanical factors may contribute to ischemic ventricular arrhythmias. GsMtx4 peptide, a selective stretch-activated channel blocker, inhibits stretch-induced atrial arrhythmias. We aimed to assess whether GsMtx4 protects against ventricular ectopy and arrhythmias following coronary occlusion in swine. First, the effects of 170-nM GsMtx4 on the changes in the effective refractory period (ERP) induced by left ventricular (LV) dilatation were assessed in 8 isolated rat hearts. Then, 44 anesthetized, open-chest pigs subjected to 50-min left anterior descending artery occlusion and 2-h reperfusion were blindly allocated to GsMtx4 (57 μg/kg iv. bolus and 3.8 μg/kg/min infusion, calculated to attain the above concentration in plasma) or saline, starting 5-min before occlusion and continuing until after reflow. In rat hearts, LV distension induced progressive reductions in ERP (35±2, 32±2, and 29±2 ms at 0, 20, and 40 mmHg of LV end-diastolic pressure, respectively, P<0.001) that were prevented by GsMTx4 (33±2, 33±2, and 32±2 ms, respectively, P=0.002 for the interaction with LV end-diastolic pressure). Pigs receiving GsMtx4 had similar number of ventricular premature beats during the ischemic period as control pigs (110±28 vs. 103±21, respectively, P=0.842). There were not significant differences among treated and untreated animals in the incidence of ventricular fibrillation (13.6 vs. 22.7%, respectively, P=0.696) or tachycardia (36.4 vs. 50.0%, P=0.361) or in the number of ventricular tachycardia episodes during the occlusion period (1.8±0.7 vs. 5.5±2.6, P=0.323). Thus, GsMtx4 administered under these conditions does not suppress ventricular ectopy following coronary occlusion in swine. Whether it might protect against malignant arrhythmias should be tested in studies powered for these outcomes.

## Introduction

Malignant ventricular arrhythmias are frequent and life-threatening complications of acute myocardial ischemia. While the mechanisms of ischemic arrhythmias are complex and not completely understood [1,2], the potential contribution of mechanical factors is progressively gaining recognition [3,4]. On the one hand, these arrhythmias are often initiated by ventricular premature beats (VPBs) originated at the ischemic border [5–7], an area of increased mechanical tension. On the other hand, the border zone modulates the distribution of wave breaks during ventricular fibrillation, regional ischemia increasing wave break incidence at this zone [8,9]. Finally, spontaneous [10–13], induced [6,14], or simulated [7] left ventricular distension has been associated with an increased arrhythmogenicity following coronary occlusion.

Mechanically-induced arrhythmias have been suppressed in different experimental preparations not involving ischemia by gadolinium or other blockers of stretch-activated ion channels (SACs) [15–19]. However, attempts to protect against ventricular arrhythmias after coronary occlusion in vivo with intravenous [11] or intracoronary [12] gadolinium have failed. While this failure might reflect the absence of a significant contribution of SACs to ischemic ventricular arrhythmias, it may also have been due in part to the limited bioavailability of gadolinium [20].

GsMtx4, a peptide isolated from tarantula venom that selectively inhibits SACs [21] and that has effectively suppressed stretch-induced atrial fibrillation in perfused rabbit hearts [22] might circumvent this limitation. Our aim was to assess whether GsMtx4 peptide, administered intravenously prior and during ischemia, would reduce ventricular ectopy and the number and severity of arrhythmias during coronary occlusion in anesthetized swine.

## Materials and Methods

The experimental protocol was approved by the Ethical Committee on Animal Experimentation of Vall d’Hebron Research Institute (ref. CEEA 25/05). GsMtx4 peptide was synthesized (21^st^ Century Biochemicals, Marlboro, MA) as previously described [23]. As determined by high-performance liquid chromatography, the purity of the linear peptide was 97.7%. After synthesis, the peptide was folded by reduction with glutathione as previously described [23].

### Experiments in isolated rat hearts

#### Experimental preparation and monitoring

These experiments were aimed to confirm that the synthesized peptide was biologically active against stretch-induced electrophysiological changes. Eight male Sprague-Dawley rats (300–350 g) were anesthetized with intraperitoneal sodium pentobarbital (100 mg/kg). Hearts were mounted into a non-recirculating Langendorff apparatus, and perfused at 10 ml/min with modified Krebs-Henseleit bicarbonate buffer equilibrated with 95% O_2_/5% CO_2_ as previously described [24]. A fluid-filled latex balloon was introduced into the left ventricle (LV) to monitor intraventricular pressure. Two hook electrodes were implanted approximately 3-mm apart over the mid anterior aspect of the LV. LV-developed pressure was calculated as the difference between LV systolic pressure and LV end-diastolic pressure (LVEDP). Perfusion pressure was continuously recorded. These signals, along with the electrogram, were acquired, digitized, and measured using a 16-channel PowerLab system (ADInstruments, Bella Vista, Australia).

#### Determination of the effective refractory period and arrhythmia inducibility and their modification by changing the loading conditions and by GsMtx4

**Fig 1:**
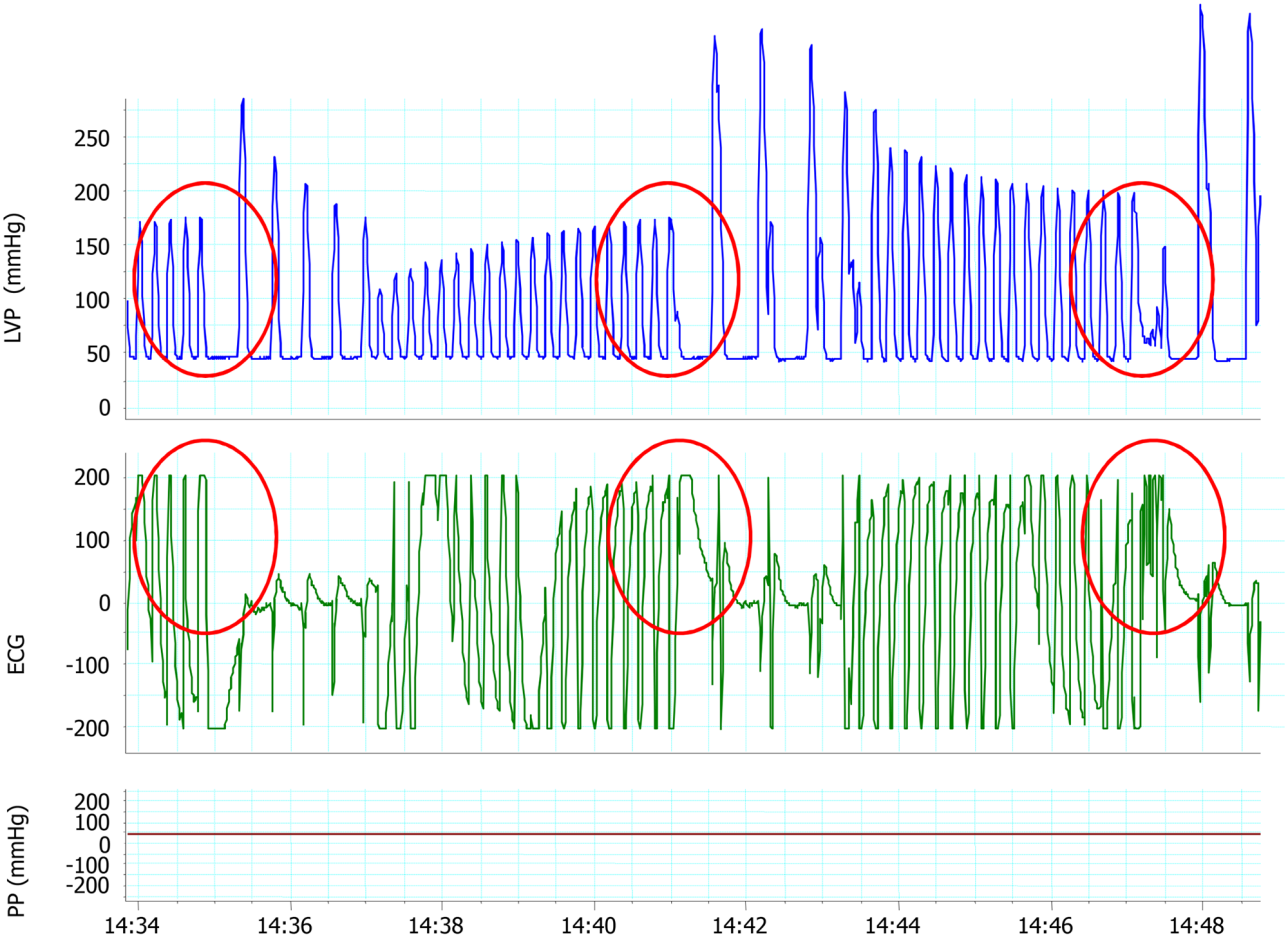
Example of the stimulation protocol in isolated rat hearts. Three consecutive stimulation cycles at left ventricular enddiastolic pressure = 40 mmHg and with progressively increasing (1 ms) coupling intervals are depicted (circles): the first extrastimulus is not conducted (effective refractory period), the second one is conducted and the third one triggers a short run of ventricular tachycardia. ECG = Electrocardiogram; LVP = Left ventricular pressure; PP = Perfusión pressure.

Hearts were paced at a 250-ms cycle length (UHS-20 stimulator, Biotronik, Berlin, Germany). Programmed bipolar stimulation was performed to determine the effective refractory period (ERP). After a drive train of 20x200 ms, a ventricular extrastimulus was introduced with an initial coupling interval of 15-ms that was progressively increased at 1-ms steps until capture was observed twice in a row. The ERP was defined as the highest coupling interval failing to capture. The stimulation protocol was continued until reaching a coupling interval of 5-ms above the ERP and the number of episodes of induced ventricular tachycardia in these 5 runs was counted and their duration recorded (Fig 1).

These measurements were performed in each heart at 3 different values of LVEDP: 0, 20 and 40 mmHg, in random order. LVEDP was modified by changing the volume of fluid in the balloon. Measurements were performed sequentially with and without GsMtx4 in the perfusate, also in a random order and in a blinded fashion. During the first set of measurements, a solution containing GsMtx4 or only the buffer was added through a lateral line, and the second set of measurements was repeated after 30-min washout by adding the complementary solution. GsMtx4 was infused at a final concentration of 170 nM, which suppressed stretch-induced atrial arrhythmias ex-vivo [22]. The 30-min washout time also proved to ensure the complete disappearance of GsMtx4 effects [22].

### Experiments in pigs subjected to coronary occlusion

#### Animal preparation

Forty-six domestic hybrid pigs of either sex (33±1 kg) were premedicated with 10 mg/kg intramuscular azaperone and anesthetized with 30 mg/kg intravenous thiopental sodium followed by a continuous infusion. We previously observed in this animal model that the dilatation of the ischemic region was associated with ventricular fibrillation or tachycardia after coronary occlusion [10–13]. Animals were intubated and mechanically ventilated. One femoral artery and vein were catheterized, a sternotomy was performed and the pericardium was opened. The mid left anterior descending artery (LAD) was dissected and surrounded by a snare, adjacent to which a Doppler flow probe was placed. Two pairs of ultrasonic crystals were inserted into the LV wall, along a plane perpendicular to the long axis of the ventricle, one pair in the myocardium to be made ischemic and the other in the lateral wall. A micromanometer-tipped catheter (Mikro-tip, Millar, Houston, TX) was inserted into the LV.

#### Experimental protocol and postmortem studies

After attaining hemodynamic stability, the LAD was occluded by tightening the snare. Animals were blindly allocated to receive GsMtx4 or saline. In the former group, a saline solution containing 57.4 μg GsMtx4/kg was administered intravenously during 2 min starting 5 min before coronary occlusion (a blood concentration of 200-nM GsMTx4 was estimated assuming a molecular weight of 4.1 KDa, a purity of 97.7% and a blood volume of 70 mL/kg). After this bolus, a maintenance infusion of 3.8 μg/kg/min was provided during the following 58 min. Fifty minutes after coronary occlusion, reperfusion was allowed for 2 hours, after which time the LAD was re-occluded. Animals were euthanized by pentobarbital overdose followed if needed by inducing ventricular fibrillation. The heart was excised and cut in slices and the mass of the ischemic region (in vivo fluorescein injection) and infarct size (triphenyltetrazolium chloride reaction) were calculated as previously described [10–13].

Two animals were excluded, one before randomization due to major bleeding during sternotomy and one after randomization due to incessant ventricular fibrillation immediately after coronary occlusion, showing the examination of the heart severe subaortic stenosis and LV hypertrophy. Thus, this series was composed of 44 valid experiments, 22 in each treatment arm.

#### Study monitoring

**Fig 2:**
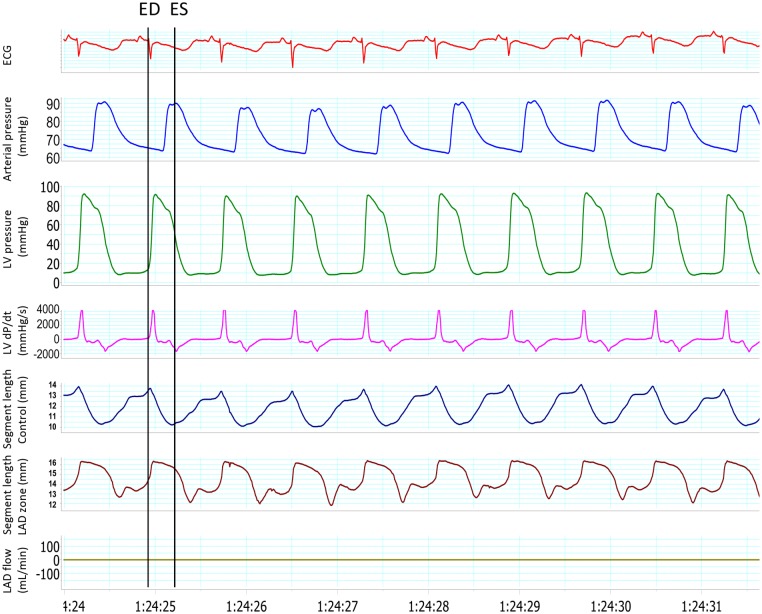
Example of monitoring in anesthetized pigs. This tracing was obtained in the ischemic period. Segment length curves clearly show an abnormal wall motion in the ischemic zone as compared to the normal motion in the control zone. ECG = Electrocardiogram; ED = End-diastole; ES = End-systole; LAD = Left anterior descending artery; LV = Left ventricular.

Serial arterial blood gases were obtained to adjust the ventilator parameters. Serum pH, sodium, potassium, and bicarbonate levels and the hematocrit value were measured at baseline and at the end of the experiment. Aortic pressure was monitored. The ultrasonic signals were analyzed with an ultrasonic dimension system (System 6/200, Triton Technology, San Diego, CA) and monitored with an oscilloscope (HM 205–3, Hameg, Frankfurt, Germany). These signals, along with an electrocardiographic lead, aortic pressure, LAD blood flow and LV pressure and its first derivative (dP/dt) were continuously recorded, digitized (ML795 PowerLab) and stored (Fig 2). If ventricular fibrillation occurred, the heart was defibrillated with 10–20 J shocks.

In 12 animals (6 per group), the ventricular ERP was determined by programmed stimulation, as previously described [13], before and after bolus administration of GsMtx4 or placebo.

#### Segment length measurements and ventricular arrhythmias

Segment length measurements were performed on the digital records as previously described [10–13]. End-diastolic and end-systolic segment lengths (EDL, ESL) and maximal segment length were measured at several time points. Systolic shortening was calculated as follows (%): (EDL–ESL)x100/EDL. EDL was expressed as a percentage of values before coronary occlusion. Systolic bulging was defined as the ratio of maximal segment length during systole and EDL of the same beat times 100%. The number of VPBs during the occlusion period was counted and the occurrence of ventricular tachycardia (≥3 consecutive ventricular beats) or fibrillation was recorded. Only the initiating ventricular beat of ventricular tachyarrhythmias was counted as a VPB. Phases IA and IB ischemic arrhythmias were defined as those occurring, respectively, before or after 10 min of coronary occlusion [25]. Ventricular fibrillation occurring in the first 3 min after reperfusion was also recorded.

### Statistical analysis

According to the frequency and variability (mean = 74, variance = 1681) of VPBs after coronary occlusion in a previous study in the same model [11], the sample size (22 animals per group) was powered to detect, with α- and ß-probabilities of 0.05 and 0.2, respectively, a 42% reduction in the number of VBPs. Statistical analysis was performed with SPSS software (Chicago, IL). Categorical variables are described as frequencies and percentages, and continuous variables as means±SE. Differences between two independent groups were assessed by the chi-square test for categorical variables and by Student t-tests for continuous variables. General analysis of variance for repeated measures was performed to assess the effect of the loading conditions on ERP and its interaction with treatment in isolated hearts and the effect of time and treatment on hemodynamics, segment length changes, and blood test results in pigs. The effect of time and GsMtx4 on VBP incidence (square-root-transformed) throughout the occlusion period was assessed with a linear mixed effect model. The effect of treatment on ventricular arrhythmia incidence was also assessed after adjusting for relevant variables using logistic regression analyses. P values <0.05 were considered significant.

## Results

### Experiments in isolated rat hearts

**Fig 3:**
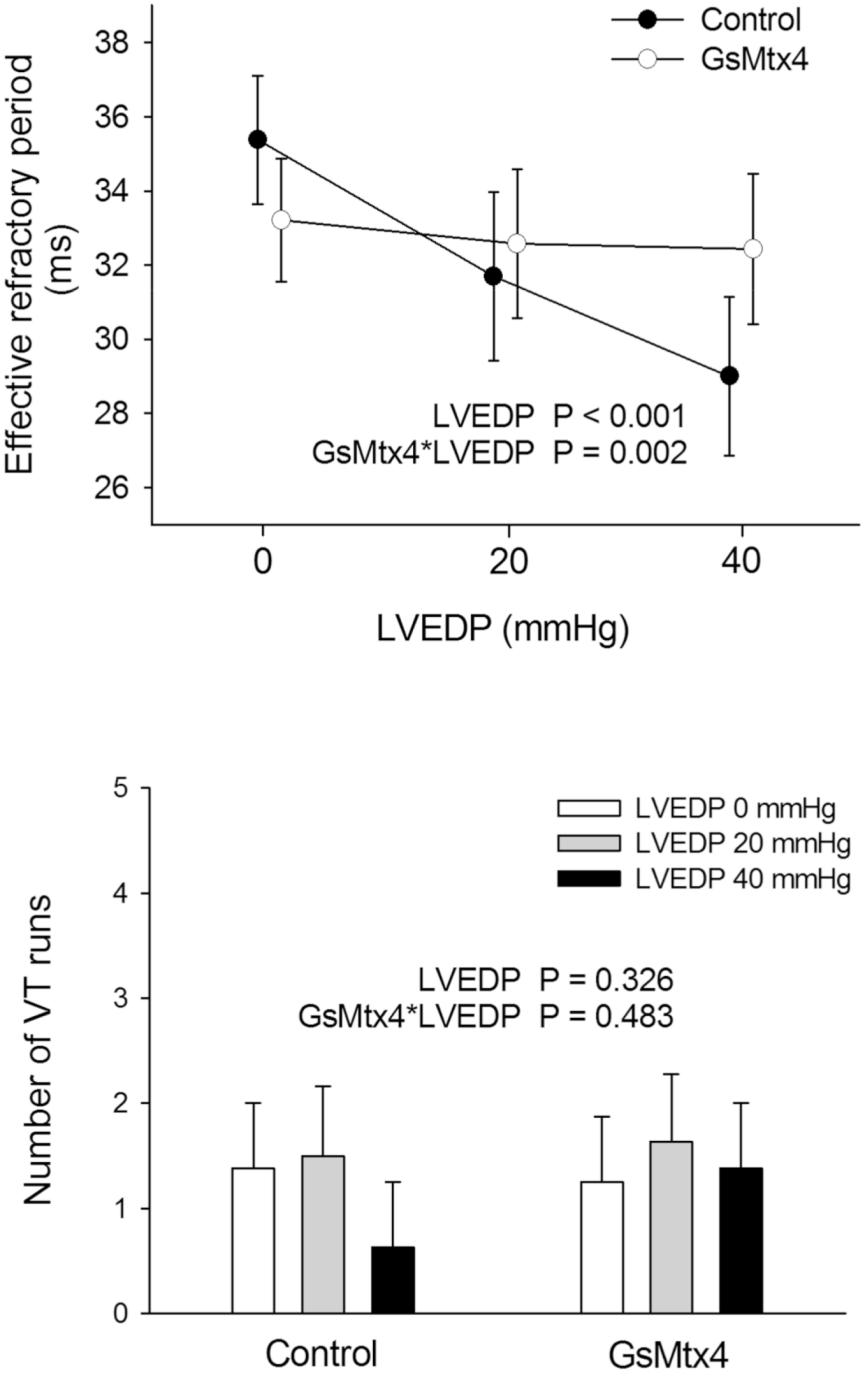
Summary of the main results of the experiments in isolated rat hearts. Top: Values of the ventricular effective refractory period in isolated rat hearts at different loading conditions and with or without GsMtx4 in the perfusate. Bottom: Number of runs of ventricular tachycardia induced by 5 stimulation protocols with coupling intervals 1 to 5 ms over the effective refractory period in isolated rat hearts. LVEDP = Left ventricular enddiastolic pressure; VT = Ventricular tachycardia.

Before programmed stimulation, LV developed pressure averaged 109±4 mmHg and perfusion pressure 64±1 mmHg, without differences among treated and untreated conditions. The results of these experiments are illustrated in Fig 3. In untreated hearts, the ventricular ERP decreased progressively as LVEDP increased (35±2, 32±2, and 29±2 ms at 0, 20, and 40 mmHg of LVEDP, respectively, P<0.001). However, when GsMtx4 was present in the perfusate, increasing the loading conditions did not affect the ERP (33±2, 33±2, and 32±2 ms, respectively, P = 0.002 for the interaction with LVEDP). Ventricular tachycardia runs in the 5 pre-defined stimulation protocols occurred with the same frequency at the 3 LVEDP values tested and their frequency was also not modified by GsMtx4. The total number of ventricular ectopic beats in these runs was also comparable at the 3 LVEDPs tested in the absence (9±5, 35±29, and 5±3, respectively) and in the presence (7±3, 8±3, and 8±3, respectively) of GsMtx4 (P = 0.569 for LVEDP, P = 0.436 for the interaction GsMtx4*LVEDP). Ventricular fibrillation was not induced.

### Hemodynamic data in anesthetized pigs

Hemodynamic data are summarized in Table 1. At baseline, the hemodynamic parameters and LAD blood flow were within the normal range and similar in both groups, and were not modified—but for a slight increase in mean aortic pressure—after infusion of GsMtx4 or saline. Coronary occlusion induced significant (P<0.001) changes consisting of an increase in heart rate and LVEDP and a reduction in mean aortic pressure. Heart rate and aortic pressure increased progressively thereafter (P<0.001) whereas LVEDP remained unchanged (P = 0.488). LAD blood flow during the reperfusion period was higher than at baseline (P<0.001). These changes were not significantly affected by treatment allocation.

**Table 1:** Hemodynamic data in anesthetized pigs.

	Baseline	Pre-CO	5-min CO	15-min CO	30-min CO	50-min CO	30-min R	1-h R	2-h R
Heart rate, bpm									
Control	83±4	84±4	88±5	91±5	96±6	103±7	117±6	118±5	117±5
GsMtx4	85±4	85±4	88±4	88±5	90±5	91±6	110±5	113±8	118±6
Mean AP, mmHg									
Control	72±3	76±4	69±3	72±3	74±4	78±4	72±3	82±4	85±3
GsMtx4	71±4	73±4	68±4	70±4	72±4	71±4	71±3	77±4	84±5
LVEDP, mmHg									
Control	7±1	8±1	15±2	15±2	16±2	18±2	13±2	17±3	13±2
GsMtx4	7±1	8±1	13±1	15±1	15±2	15±2	15±2	14±1	13±2
Mean LAD flow, ml/min									
Control	16±2	--	--	--	--	--	26±3	31±4	31±4
GsMtx4	15±2	--	--	--	--	--	23±2	23±3	27±4

Values are means ± SE. AP = Aortic pressure, LAD = left anterior descending artery, LVEDP = Left ventricular enddiastolic pressure, CO = coronary occlusion, R = reperfusion. No significant between-group differences were observed (two-way repeated-measures analysis of variance); significance values of within-group changes are described in the text.

### Segment length changes, blood tests and post-mortem studies in anesthetized pigs

Segment length changes are summarized in Table 2. At baseline, EDL averaged 12.5±2.5 mm in the control zone and 13.3±2.2 mm in the LAD region. EDL and systolic shortening were not affected by the infusion of GSMtx4 or saline before ischemia. In the control zone, EDL increased slightly after occlusion and systolic shortening remained unchanged. EDL values returned to baseline throughout the experiment, without between-group differences, whereas systolic shortening experienced a minor reduction after reperfusion in controls but not in treated animals (P = 0.02). In the LAD region, coronary occlusion induced a marked increase in EDL and abolished systolic shortening (both P<0.001). EDL remained stable during ischemia and declined after reperfusion to values below baseline, whereas systolic shortening persisted severely depressed throughout the experiment. No significant interactions were found between segment length changes in the LAD region and treatment allocation. The percentage of animals with greater (above the median) percent reduction in EDL in the reperfused zone 30 min after reflow, a surrogate of hypercontracture [26] was similar in treated and untreated animals (42.9 vs. 57.9%, respectively, P = 0.342).

**Table 2:** Segment length changes in anesthetized pigs.

	Baseline	Pre-CO	5-min CO	15-min CO	30-min CO	50-min CO	30-min R	1-h R	2-h R
Nonischemic region									
EDL, % of baseline									
Control	100	101.3±0.5	103.5±0.8	103.6±1.0	103.4±1.1	103.0±1.0	98.5±0.9	100.0±0.8	99.7±1.0
GsMtx4	100	100.5±0.4	102.4±0.6	102.8±0.5	103.1±0.8	103.2±0.6	101.8±1.1	101.5±1.0	100.6±0.7
Systolic shortening, %									
Control	22.9±1.2	23.3±1.2	23.8±1.3	24.0±1.3	23.8±1.5	23.8±1.3	22.7±1.3	23.4±1.1	21.4±1.3
GsMtx4	22.2±1.0	22.0±1.0	22.6±1.2	22.7±1.2	23.4±1.1	23.9±1.1	23.8±1.3	23.5±1.3	22.4±1.5
Ischemic region									
EDL, % of baseline									
Control	100	101.1±0.7	111.0±1.0	111.3±1.0	110.6±1.1	109.8±1.4	84.0±2.7	86.6±2.6	89.2±2.7
GsMtx4	100	100.5±0.4	110.7±1.5	112.0±1.5	112.1±1.5	109.2±1.5	86.4±3.1	87.9±3.0	88.2±3.2
Systolic shortening, %									
Control	28.3±1.4	28.2±1.3	0.2±0.6	-1.1±0.7	-1.4±0.6	-0.3±0.4	1.7±0.9	1.4±0.7	-0.1±0.8
GsMtx4	29.7±1.2	28.4±1.8	-0.1±0.9	-1.4±0.9	-1.3±0.9	0.5±0.7	2.6±1.0	2.3±1.1	1.2±1.1
Systolic bulging, %									
Control			4.0±0.5	4.3±0.5	4.6±0.4	2.9±0.4	3.3±0.7	3.5±0.6	4.7±0.8
GsMtx4			6.0±0.7	6.0±0.7	5.6±0.6	3.5±0.4	3.2±0.9	3.6±0.8	4.0±0.9

Values are means ± SE. CO = coronary occlusion, EDL = Enddiastolic length, R = reperfusion. Significance values of between-group and within-group changes (two-way repeated-measures analysis of variance) are described in the text.

Table 3 summarizes blood test results. All variables remained stable throughout the experiment but for an increase in potassium levels, with no effect of GsMtx4 on any of them.

**Table 3:** Blood tests in anesthetized pigs.

	Control	GsMtx4	P value (time/GsMtx4)
Baseline/End of experiment			
pH	7.49±0.02 / 7.50±0.01	7.46±0.02 / 7.51±0.02	0.127 / 0.235
Na^+^, mmol/L	138.2±0.6 / 139.5±1.0	138.4±0.6 / 138.6±0.5	0.164 / 0.316
K^+^, mmol/L	3.54±0.10 / 4.03±0.12	3.17±0.09 / 3.87±0.14	<0.001 / 0.266
HCO3^-^, mmol/L	31.2±1.0 / 32.0±0.6	28.3±0.7 / 30.0±0.9	0.065 / 0.520
Hematocrit, %	24.8±1.0 / 25.8±1.2	25.6±1.0 / 25.8±0.9	0.173 / 0.278

Values are means ± SE. P values reflect the effect of time and of GsMtx4 (interaction term, two-way repeated-measures analysis of variance).

The size of the area at risk was comparable in animals receiving GsMtx4 or saline (19.2±2.8 and 18.9±2.7% of ventricular mass, respectively, P = 0.922). Infarct size was also not significantly different in both groups, either considered as absolute infarct mass (13.7±1.6 vs. 16.1±1.2 g, respectively, P = 0.235), as a percentage of ventricular mass (9.1±1.0 vs. 10.6±0.6%, respectively, P = 0.208) or as a percentage of the area at risk (54.2±6.1 vs. 65.4±5.2%, respectively, P = 0.166).

### Effective refractory period and ventricular arrhythmias in anesthetized pigs

At baseline, ventricular ERP averaged 255±10 ms in controls and 277±8 ms in animals assigned to receive GsMtx4 (P = 0.134). ERP was not modified by bolus administration of either saline or GsMtx4 (286±16 and 283±8 ms, respectively, P = 0.149 for time, P = 0.337 for the interaction GsMtx4*time). Baseline ERPs were similar in animals experiencing or not ventricular fibrillation (245±35 vs. 270±6 ms, respectively, P = 0.604) or ventricular tachycardia or fibrillation (260±14 vs. 270±8 ms, respectively, P = 0.515) during the ischemic period.

**Fig 4:**
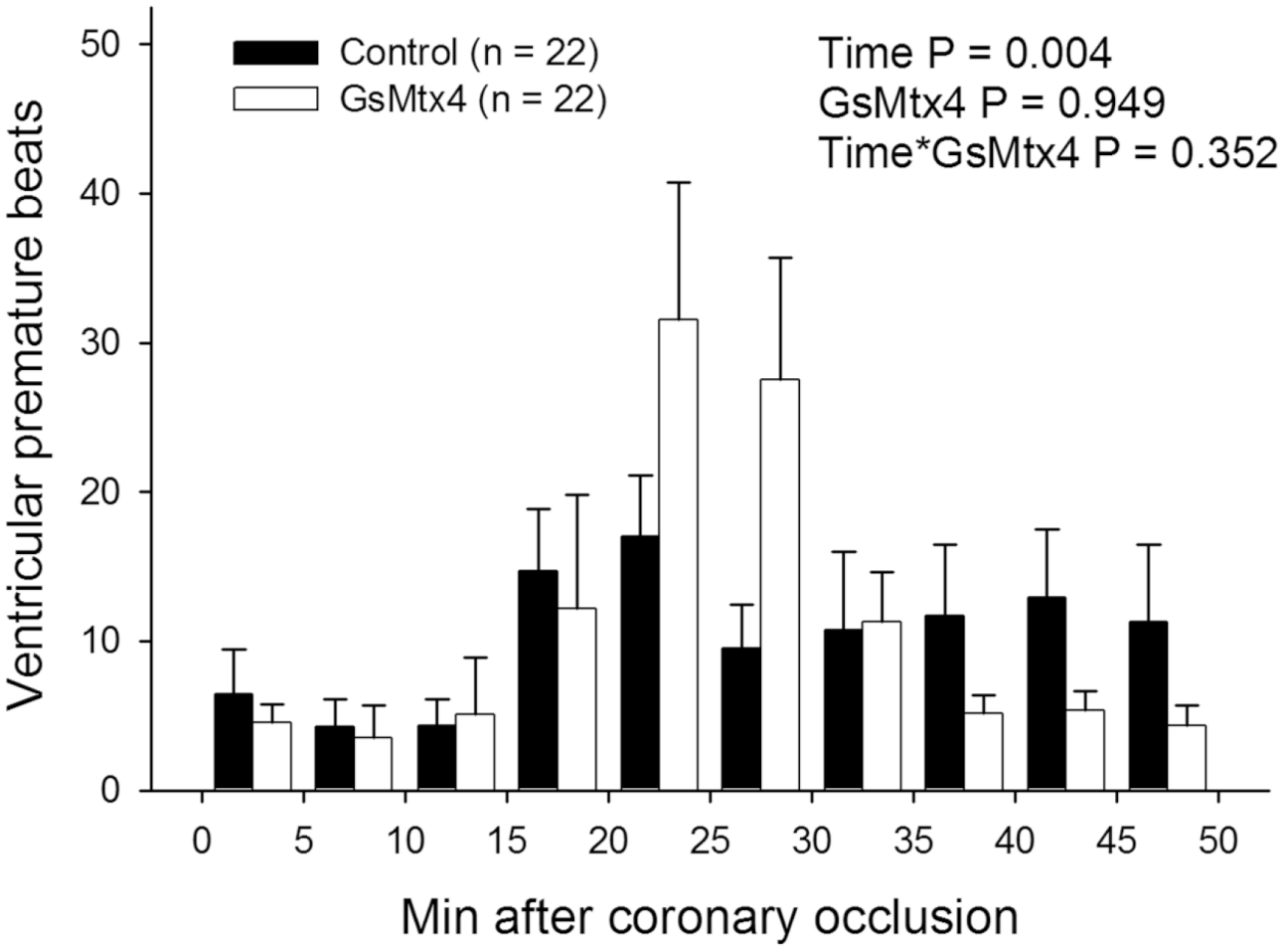
Distribution of ventricular premature beats during the ischemic period in treated and untreated pigs. P values obtained with a linear mixed effect model after square-root transformation of data.

The distribution of VPBs during coronary occlusion is depicted in Fig 4. The mean number of VPBs was comparable in animals treated with GsMtx4 and in those receiving saline, both in the IA phase (8±3 vs. 11±5, respectively, P = 0.622), in the IB phase (102±27 vs. 92±20, respectively, P = 0.771) or overall (110±28 vs. 103±21, respectively, P = 0.842).

**Fig 5:**
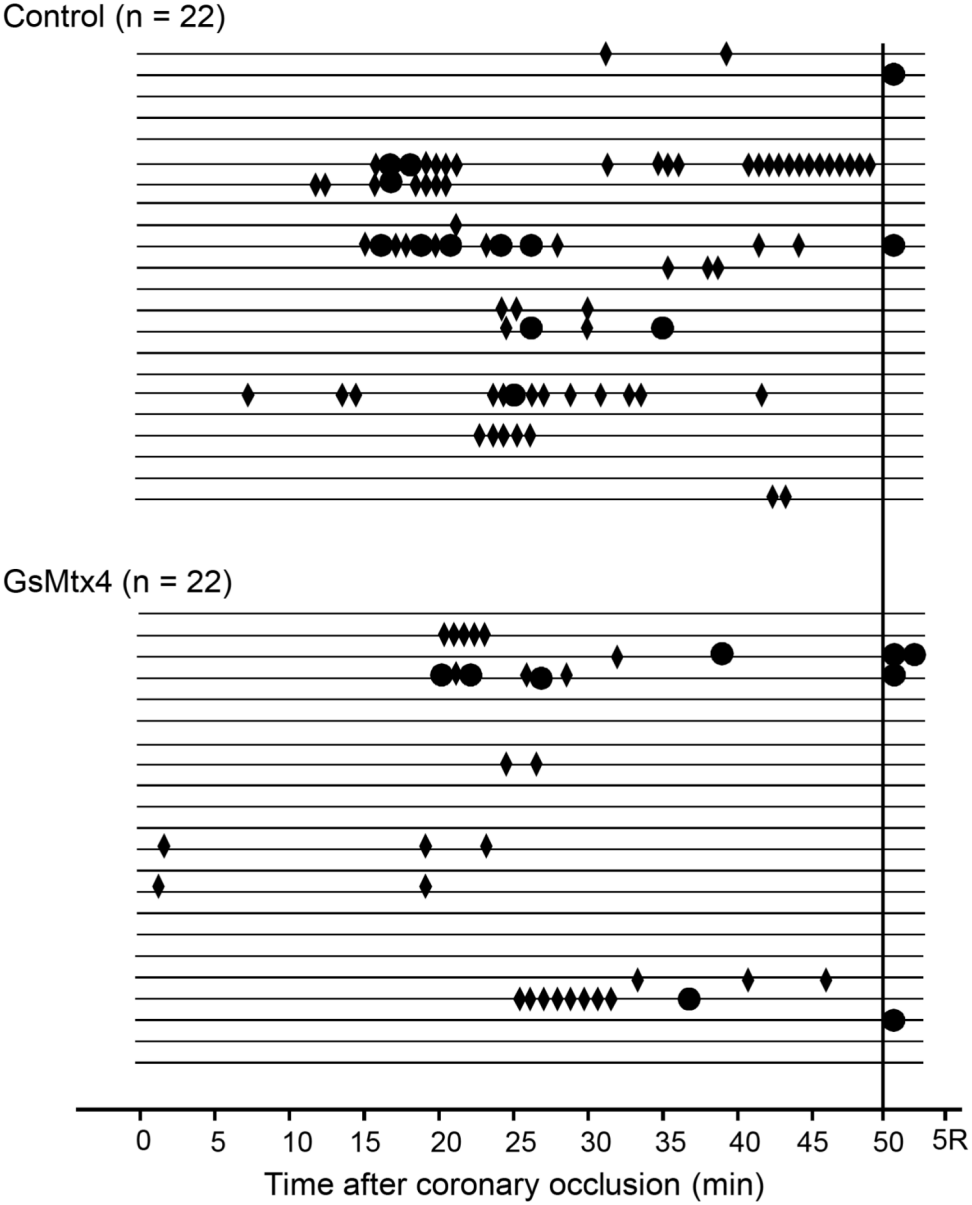
Distribution of episodes of ventricular tachycardia (rhombi) or fibrillation (circles) during ischemia and initial reperfusion in both groups. Each line represents an animal. In cases with a very high density of ventricular tachycardia episodes not all of them could be depicted. R = Reperfusion.

Fig 5 illustrates the occurrence of ventricular tachyarrhythmias during ischemia and immediately after reperfusion. Eight animals had ventricular fibrillation during ischemia, the first episode occurring 25±9 min after coronary occlusion. In comparison to controls, treated animals did not show significant reductions in the incidence of ischemic ventricular fibrillation (13.6 vs. 22.7%, respectively, P = 0.696), ventricular tachycardia (36.4 vs. 50.0%, respectively, P = 0.361), ventricular tachycardia or fibrillation (36.4 vs. 50.0%, respectively, P = 0.361), in the number of ventricular tachycardia episodes during coronary occlusion (1.8±0.7 vs. 5.5±2.6, respectively, P = 0.323), or in the total duration of ventricular tachycardia (14±6 vs. 38±19 ventricular ectopic beats per animal, respectively, P = 0.384). The duration of individual episodes of ventricular tachycardia was comparable in both groups (9±3 vs. 7±2 beats, respectively, P = 0.657). GsMtx4 treatment was also not significantly associated with the incidence of ventricular fibrillation (33.3 vs. 56.3%, respectively, P = 0.632) or ventricular tachycardia or fibrillation (37.5 vs. 57.1%, respectively, P = 0.658) in the subset of animals with greater ischemic distension (increase in EDL above the median) or in those with an ischemic area size above the median (25.0 vs. 52.9%, P = 0.586, and 36.4 vs. 60.0%, P = 0.518, respectively). In multivariable analyses, treatment with GsMtx4 was not significantly associated with ventricular arrhythmia occurrence after adjusting by the magnitude of ischemic expansion or by the size of the ischemic region in the overall sample. If only arrhythmias occurring during the first 30 min of ischemia were considered, to eliminate the influence of a hypothetical dissipation of GsMtx4 effect in the ischemic region, the results also would not change significantly (ventricular fibrillation incidence: 4.5 vs. 22.7%, respectively, P = 0.185; ventricular tachycardia or fibrillation incidence: 27.3 vs. 36.4%, respectively, P = 0.517). Five animals had ventricular fibrillation immediately after reperfusion (14.3 vs. 9.1%, respectively, P = 0.664).

Compared with the remaining animals, those presenting ventricular fibrillation during ischemia had similar increase in EDL in the ischemic zone 15 min after coronary occlusion (114.7±2.7 vs. 111.0±0.9% of baseline, respectively, P = 0.117), baseline potassium values (3.6±0.2 vs. 3.5±0.1 mmol/l, respectively, P = 0.679), size of the ischemic region (18±1 vs. 19±2% of ventricular mass, respectively, P = 0.781), and infarct size (58±8 vs. 60±5% of the ischemic region, respectively, P = 0.854).

## Discussion

The primary objective of this study was to assess the effect of intravenous administration of the selective SAC blocker GsMtx4 peptide on ischemic VPBs in swine. Compared with controls, treated animals had virtually the same number of VPBs, indicating that GsMtx4, administered under these conditions, does not suppress ventricular ectopic activity following coronary occlusion in swine. The results are compatible with some protective effect against ventricular tachycardia or fibrillation, but the study was not powered for these outcomes.

### Loading conditions, ventricular refractoriness and arrhythmia inducibility in isolated rat hearts

LV dilatation decreased LV-ERP in isolated rat hearts, which concurs with previous observations in rabbit ventricles [27,28] or atria [22]. Previously, GsMTx4 non-significantly attenuated the stretch-induced decrease in ERP in isolated rabbit atria [22]. In the present study, GsMtx4 significantly altered the stretch-dependence of the ERP in isolated rat hearts, indicating that the synthetized peptide was biologically active.

LV dilatation was not associated with the incidence or duration of ventricular tachycardia episodes induced by the introduction of a single extrastimulus after continuous ventricular pacing for the determination of LV-ERP, nor did it facilitate ventricular fibrillation occurrence. These results are at variance with previous observations in rabbit ventricles [27,28] but concur with studies in canine [29]. Anyway, they made that the lack of association of GsMtx4 with ventricular tachycardia occurrence observed in our experiments was predictable.

### Effects of SAC blockade on ventricular arrhythmias following coronary occlusion

Intravenous GsMtx4 did not affect the ERP in unloaded ventricles and did not suppress ventricular ectopic activity after coronary occlusion in swine. A positive result would have been expected according to previous studies suggesting a significant role of mechanical factors in the genesis of VBPs during regional ischemia. In this respect, it was described that increasing the loading conditions may affect the action potential duration after coronary occlusion [14]. In isolated, swine hearts it was shown that a substantial number of VPBs originate close to the ischemic border [5,6] and that their number is augmented by increasing LV volume [6]. Finally, modeling experiments of rabbit ventricles indicated that mechanical activity contributes to the origin of VBPs in the ischemic border during regional ischemia [7]. The present results, however, are in line with previous studies in the same model in our laboratory showing that the dilatation of the ischemic region after coronary occlusion was not related to the number of VPBs [10–13]. The reasons for the lack of association between the increase in EDL and VPB incidence following coronary occlusion in vivo are unclear. Besides the multiplicity of factors involved in arrhythmogenesis during ischemia, a possible explanation is that the increase in EDL reflects a stable, passive dilatation of the ischemic region more likely to enhance the vulnerability of the substrate than to act as a trigger. In this respect, in earlier studies demonstrating that stretch induces VPBs, mechanical stress was applied in abrupt and very short pulses during early diastole [17,30], a setting very different from the stable expansion measured by EDL increase in our model. Supporting in part this interpretation, the effect of increasing the loading conditions on the incidence of ventricular arrhythmias during regional ischemia in isolated hearts was comparably greater for complex arrhythmias than for isolated VPBs [6]. The magnitude and the direction of the differences in VPB density between treated and untreated animals were not homogeneous throughout the ischemic period. However, given the variability in VBP occurrence and that the effect of the treatment was not statistically significant, these differences over time may have just been the result of chance.

Our study was not powered to evaluate the effects of GsMtx4 on ventricular tachycardia or fibrillation, although the results are compatible with some protective effect of GsMtx4. In line with this, increasing LV wall stress has augmented the incidence of ventricular tachycardia or fibrillation in isolated, swine hearts [6] and regional ischemic dilatation has been associated with spontaneous ventricular tachycardia or fibrillation after coronary occlusion in anesthetized swine independently of any changes in ventricular ectopic activity [10–12]. We recently observed that distension of the ischemic region after coronary occlusion is associated with an increased susceptibility to ventricular fibrillation by programmed electrical stimulation in swine [13]. All these results strongly suggest a direct contribution of mechanical factors to the substrate of ventricular tachycardia or fibrillation during regional ischemia. This contribution is further supported by recent modeling experiments of rabbit ventricles [7] and is consistent with observations that stretch modulates ventricular fibrillation characteristics in ischemic and non-ischemic conditions [8,31].

The paucity of events made it not possible to assess the effect of GsMtx4 on post-reperfusion ventricular fibrillation, although a significant effect was not expected.

### Other effects of GsMtx4 in vivo

GsMtx4 appeared to be well tolerated in vivo since it did not influence hemodynamics or segment length changes and had no effect on the laboratory parameters tested. Since this peptide reduces intracellular calcium accumulation in response to stretch [32] and targeting calcium overload has proven effective against ischemia-reperfusion injury [33], a reduction of lethal myocardial injury after transient coronary occlusion by GsMtx4 might be expected. As administered in the present study, however, neither hypercontracture nor infarct size were significantly reduced, although a protective effect at larger doses or with a more prolonged infusion throughout the reperfusion period—since the study endpoint were ischemic arrhythmias the infusion was stopped 3 min after reflow—cannot be excluded.

### Methodological considerations and limitations

To the best of our knowledge, this is the first study that has tested the effects of intravenous GsMtx4 in vivo. The dose of GsMtx4 was aimed to attain a plasma concentration above that demonstrated to protect against stretch-induced arrhythmias in isolated hearts [22]. However, the optimal dose and regime of administration of GsMtx4 to block SACs in vivo have not been established and we cannot rule out that the results would have been different with a different dose or protocol. In this respect, because of the lack of established models of acute stretch-induced electrophysiological changes in vivo [34], the biological effect of GsMtx4 was assessed in isolated rat hearts and not in the pig model, which represents a limitation. In addition, spontaneous ventricular wall distension during regional ischemia might cause a weaker SAC activation than inflating a balloon in the LV, and this might have contributed to the different responses to GsMtx4 in both models in our study. Finally in this regard, given that there are structural differences between atrial and ventricular myocardium, the concentrations of GsMtx4 necessary to block SCAs in the atria and in the ventricles might not be the same, although in our study 170 nM effectively suppressed stretch-induced changes in ventricular ERP.

GsMtx4 reached the ischemic area before ischemia but only the outer side of the ischemic border was infused after coronary occlusion. Given that GsMtx4 effects last approximately 20 min in isolated hearts after washout [21,22], effective SAC blockade inside the ischemic region during the second half of the ischemic period cannot be guaranteed. Direct infusion of the peptide inside the LAD region during coronary occlusion might have overcome this limitation. However, we chose systemic administration because a significant number of ischemic VPBs originate at the outer side of the ischemic border [6] and also because this regime is more clinically relevant than intracoronary infusion. Anyway, it seems unlikely that this limitation had influenced the lack of effect of GsMtx4 on ventricular ectopic activity given that it was observed similarly in earlier or later phases of ischemia.

As mentioned before, another limitation is that our study was not powered to detect a protective effect against ventricular tachycardia or fibrillation. For such an objective, a very large sample size (150 animals per group to detect a reduction from 25 to 12.5% in the incidence of ischemic ventricular fibrillation, or 58 animals per group to detect a reduction from 50 to 25% in the combined incidence of ventricular tachycardia or fibrillation) would be required. Although the incidence of arrhythmias tended to be higher in animals with greater ventricular distension or with a larger ischemic area, it also was not significantly reduced by GsMtx4 in these subgroups or after controlling for these variables in multivariable analyses in the overall sample. In addition, the incidence of ventricular fibrillation was lower than expected [10–12]. The fact that the magnitude of expansion of the ischemic region was somewhat lower than in previous studies in the same model that used the same experimental setting and segment length measurement methodology [10–12] may have underlain in part this observation and may explain that in the present series the ischemic increase in EDL was not significantly associated with ventricular fibrillation occurrence. Although the size of the ischemic region and serum potassium levels are well-known predictors of ischemic malignant arrhythmias, their values were quite homogeneous in our experiments and it is not surprising that they were also not significantly associated with these arrhythmias.

## Conclusions

GsMtx4, administered under the present conditions, does not suppress ventricular ectopic activity following coronary occlusion in swine. The results cannot rule out some protective effect against ventricular tachycardia or fibrillation. Given the enormous impact of malignant ventricular arrhythmias in patients with acute myocardial infarction, this hypothesis should be tested in studies powered for these outcomes.
